# Structural and electronic properties of oligo- and polythiophenes modified by substituents

**DOI:** 10.3762/bjnano.3.101

**Published:** 2012-12-27

**Authors:** Simon P Rittmeyer, Axel Groß

**Affiliations:** 1 Institute of Theoretical Chemistry, Ulm University, Albert-Einstein-Allee 11, D-89069 Ulm, Germany

**Keywords:** band gaps, conducting polymers, density functional theory calculations, molecular electronics, oligothiophenes

## Abstract

The electronic and structural properties of oligo- and polythiophenes that can be used as building blocks for molecular electronic devices have been studied by using periodic density functional theory calculations. We have in particular focused on the effect of substituents on the electronic structure of thiophenes. Whereas singly bonded substituents, such as methyl, amino or nitro groups, change the electronic properties of thiophene monomers and dimers, they hardly influence the band gap of polythiophene. In contrast, phenyl-substituted polythiophenes as well as vinyl-bridged polythiophene derivatives exhibit drastically modified band gaps. These effects cannot be explained by simple electron removal or addition, as calculations for charged polythiophenes demonstrate.

## Introduction

Since the first report about the electrical conductivity of doped polyacetylene (PA) in 1977 [1], significant efforts have been spent in studying organic polymers as an alternative to common inorganic semiconducting materials [2], as they can, e.g., form supramolecular architectures on surfaces [3–4] that can serve as building blocks in molecular electronics or can be used in the future solar-energy technology [5]. Although the electrical conductivity of well-prepared PA is nearly the same as for copper [6], its technical applications are very rare due to its instability towards air and humidity [7]. Searching for more stable compounds, thiophene-based materials turned out to be promising candidates, and thus, they have gained considerable attention during the past 20 years [6,8].

Like PA, nanosized polythiophene (PTp) shows a diffuse widespread conjugated π-system [8]. Consequently, removing an electron from the highest occupied polymer orbital or adding an electron to the lowest unoccupied orbital is relatively easy [9]. In a chemist’s terminology one might call these processes redox reactions, whereas from a physicist’s point of view one would more likely call them n- and p-doping, respectively, to stress the analogy to the doping processes in traditional semiconducting materials such as silicon. Hence, neutral polymers, which usually show semiconducting or insulating properties, can transform into highly conductive compounds with a metal-like behavior.

The advantages of these *synthetic metals* are obvious. On the one hand they are nearly as conductive as metals but on the other hand they are as light and durable as plastics [10]. Furthermore, especially in the case of PTp, the doping processes causing the high conductivity of polymers are highly reversible [9]. This offers the opportunity to switch between conducting and insulating properties very easily and opens a broad field of application in the area of micro- and optoelectronics, e.g., as organic transistors, photoresistances or polymer light-emitting diodes (LEDs) [11]. In particular, thiophene-based organic solar cells have shown remarkable efficiency [5,8]. Nevertheless they are still relatively cheap in production [12].

For all these applications, the particular electronic structure of polymers is crucial. In this regard, a directed manipulation of the band gap to tailor the electronic properties is very desirable. Considering the significant potential of organic chemistry at synthesizing and manipulating compounds, there is definitely a demand for a better understanding of how the electronic structure of compounds such as PTp can be manipulated by using these tools. There have been already several studies addressing the electronic structure of thiophenes with electronic structure methods [13–20]. In these computational studies, typically oligothiophenes of varying size have been considered based on density functional theory (DFT), and the properties of polythiophenes have been derived by using scaling relations [21].

Here, we focus on the modification of the electronic properties of oligo- and polythiophenes by substituents based on periodic DFT calculations. Hence, we are able to address oligo- and polythiophenes within the same computational method so that no scaling relations have to be invoked. Our aim was in particular to determine the influence of different substituents on the electronic structure and especially on the band gap of thiophene-based polymers, as it is known that there is a close relationship between the geometrical structure and the physical properties of conductive polymers [22].

As a starting point, we first considered thiophene monomers and dimers and then compared their properties to those of infinite chains of thiophene, which can also act as a model for macrocyclic systems, namely cyclothiophenes [23]. As substituents we considered both singly bonded substituents, such as methyl, amino or nitro groups, as well as phenyl-like substituents. In addition, we studied vinyl-bridged polythiophene derivatives. Finally, we also addressed charged polythiophenes in order to model doped systems and to check whether the modified electronic properties can simply be regarded as effects resulting from band filling or band emptying.

## Methods

Our calculations are based on the periodic DFT code implemented in the Vienna Ab initio Simulation Package (VASP) [24–25]. Exchange and correlation effects were treated in the generalized gradient approximation (GGA) by using the Perdew–Becke–Ernzerhof (PBE) functional [26], which gives a reliable description of intramolecular properties [27–28]. Dispersion corrections [29] are not necessary since we are not concerned with intermolecular interaction or adsorption of the aromatic molecules [30–31]. The ionic cores were represented by projector augmented wave (PAW) potentials [32] as constructed by Kresse and Joubert [33]. The electronic one-particle wave functions were expanded in a plane-wave basis set up to a cutoff energy of 400 eV, which was checked for convergence.

All geometrical optimizations were carried out by using the conjugated gradient algorithm implemented in VASP. Molecules were geometrically optimized by using a sufficiently large unit cell in the supercell approach and one *k*-point. In contrast, the polymers were described as one-dimensional infinite chains with a 7 *×* 1 *×* 1 *k*-point sampling to replace the integration over the one-dimensional first Brillouin zone. *k*-Point convergence was carefully checked. When optimizing the polymer structure, both the geometric structure within the unit cell as well as the width of the unit cell were optimized as the latter correlates directly with the intercellular bond length.

For molecules, calculations concerning the density of states (DOS) were carried out at the Γ point with a Gaussian smearing (σ = 0*.*01 eV). For polymers, in contrast, a grid of 29 *×* 1 *×* 1 Γ-centered *k*-points and linear tetrahedron smearing with Blöchl corrections [34] were used. Geometrically optimized structures were taken as a basis for all of these calculations. Polymers of different oxidation states were modeled by changing the number of electrons per unit cell. In order to preserve the electric neutrality of the cell, a compensating background charge is generated by default.

As we are interested in the HOMO–LUMO gap of oligothiophenes and the band gaps of polythiophenes, we have to be concerned with the well-known deficiency of DFT using current-day GGA exchange–correlation functionals to reproduce the correct magnitude of band gaps. The calculated band structure can be improved by including self-energy corrections. However, including such corrections basically just affects the distance between valence and correction band, the shape and *k*-point dependence of valence and conduction bands remain more or less unchanged [35]. Furthermore, the more costly time-dependent DFT methods also do not necessarily yield better results [21]. In addition, hybrid functionals, which apparently work well for thiophenes [16], still require a significant computational effort in plane-wave codes such as VASP. As we are mainly interested in trends in the local density of states depending on the choice of the substituent, GGA-DFT calculations should be sufficient to reproduce these trends. However, one has to be aware that all absolute values of HOMO–LUMO and band gaps reported in this work are severely underestimated. As for the Fermi energy, it is throughout this work defined as the top of the valence band for polythiophenes with a band gap, and as the energy of the highest occupied state for periodic systems without a band gap.

## Results and Discussion

### Unsubstituted oligo- and polythiophenes

As a first step and as a reference, we determined the properties of unsubstituted oligo- and polythiophenes. All oligomers were modeled by using a sufficiently large box in three dimensions to avoid intermolecular interaction due to the use of a periodic DFT code. Note that in any polymer material the molecules are not isolated. However, there is no true chemical interaction between the molecules such that it is very likely that the electronic and structural properties of the oligo- and polythiophenes are not substantially modified by the presence of weakly interacting neighboring thiophenes.

For the unsubstituted monomer (thiophene, Tp), experimental geometric parameters obtained by Bak et al. [36] were reproduced quite well. Small deviations from experimental values concerning the dihedral angle were observed on modeling the dimer (2,2’-bithiophene, BTp): Calculations predicted a dihedral angle of 17*.*5° with a very flat rotational potential for angles from 0° to 30° whereas Almenningen et al. obtained an angle of about 34° using gas-phase electron diffraction [37]. There are known problems when using GGA-DFT to compute rotational barriers especially for conjugated systems [38], but there is definitely a flattening effect of a growing chain length as the trimer (2,5-bis(thiophen-2-yl)thiophene, TTp) was predicted to show a totally flat structure. This should be due to the extended π-system and, hence, definitely agrees with expectations. Regarding HOMO–LUMO gaps for the unsubstituted oligomers listed in Table 1, the previously mentioned problem of GGA-DFT when it comes to bandgaps is obvious. The calculated values are about 1 eV smaller than those measured by Diaz et al. [39]. Yet, the trend that the width of the HOMO–LUMO gap decreases with increasing size of the oligomer is reproduced by the calculations.

**Table 1:** Calculated HOMO–LUMO gaps for thiophene oligomers (in eV) compared with experimental values obtained by Diaz et al. [39].

	calculations	experiment

monomer (Tp)	4.49	5.37
dimer (BTp)	2.93	4.12
trimer (TTp)	2.21	3.52

**Figure 1:**
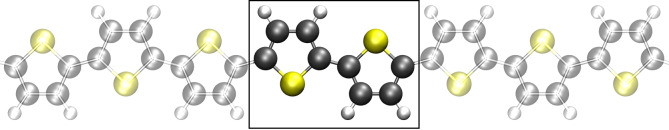
Considered structure of polythiophene (PTp). The frame indicates the unit cell used in the calculations, which contained two thiophene rings connected at their respective *α*-positions.

The polymer PTp was modeled as a one-dimensional chain, which was separated by sufficiently large distances from its periodic images perpendicular to the chain as to avoid any sizable interaction between them. As shown in Figure 1 the unit cell contained two thiophene rings. We also modeled a unit cell that contained four rings, but neither structural nor electronic parameters differed from the results for the two-ring cell.

Our calculations predict PTp to form a totally planar structure as was already calculated for the trimer. This confirms the already mentioned flattening effect of a growing chain length also found in DFT calculations for other large oligomers [27,31]. It also agrees with the results of Azumi et al. [40], who found a planar structure for the crystalline penta- and heptamer by X-ray diffraction. The calculated bond lengths are the same as in the middle ring of TTp and fit quite well to the experimental values for the heptamer [40]. This definitely justifies our ansatz to approach the polymer through smaller molecules.

**Figure 2:**
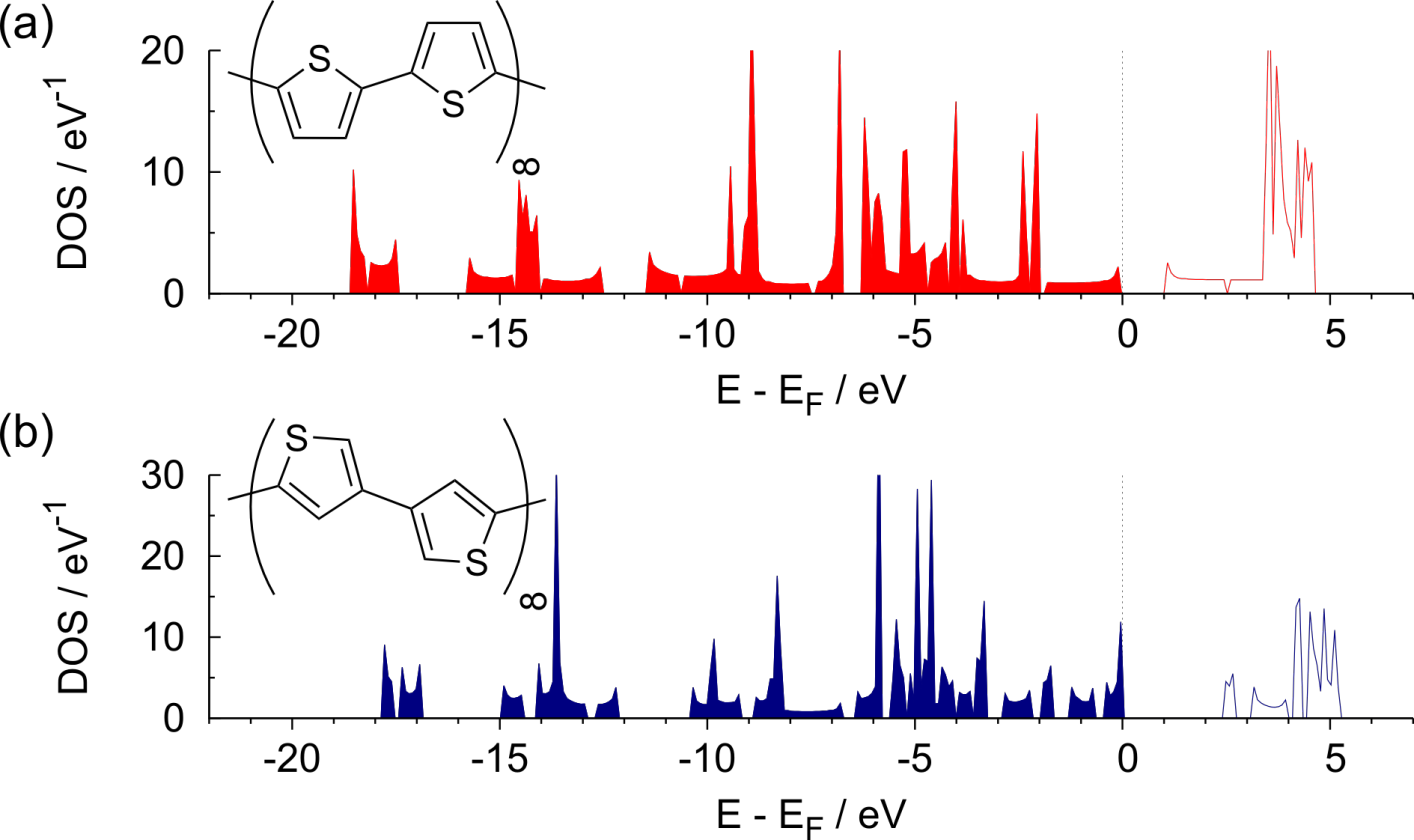
Structure and density of states for (a) PTp and (b) α,β-PTp.

Regarding the electronic structure of PTp (see Figure 2a), we obtained a band gap of 1*.*2 eV. Again, the tendency of DFT to underestimate band gaps is obvious as the calculated value is about 60% of the experimental value of 2.0 eV [41]. One might ask whether modeling linear polymers as a planar chain of infinite length could be an additional source of error in comparison with experimental values that were obtained for large, but finite and, most likely, twisted polymers. But as there are known saturation effects for electronic properties in PTp when it comes to chains consisting of 10–12 rings [42–43], this should not be a source of additional errors.

**Figure 3:**
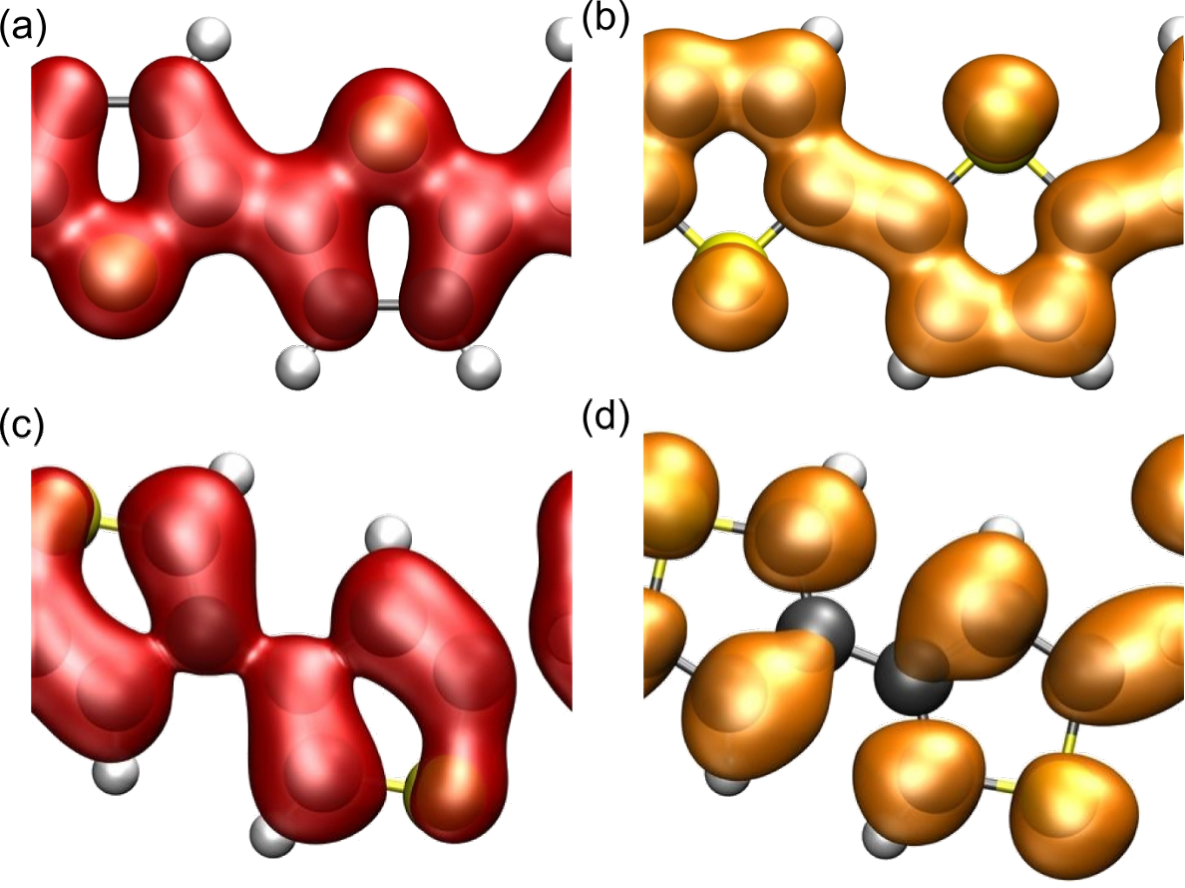
Electronic density isosurfaces (ρ(*r*) = 0*.*01 *e/*Å^3^) of the highest occupied crystal orbital (HOCO, red) and the lowest unoccupied crystal orbital (LUCO, orange) for PTp (a,b) and α,β-PTp (c,d).

In principle, there is a second possibility to build up a polymer from thiophene monomers. Instead of connecting the individual rings at their respective α-positions (2,5-connection) they can be coupled in an alternating 2,5/3,4-connection. We also modeled such an α,β-PTp system; the corresponding structure is illustrated in the inset of Figure 2b. Note that modeling a polymer consisting of exclusively 3,4-connected thiophene monomers with a two-ring unit cell is not possible because of steric hindrance. Figure 2 compares the density of states for PTp and α,β-PTp. Obviously, there is a considerable difference in the band gap of both isomers. As already mentioned, for PTp we obtained a value of 1*.*2 eV, whereas for α,β-PTp the calculated band gap of 2*.*5 eV is twice as large. This difference is most probably due to a less effective conjugation between the single-ring systems in α,β-PTp compared to PTp. As shown in Figure 3, for PTp the highest occupied crystal orbital (HOCO) as well as the lowest unoccupied crystal orbital (LUCO) are delocalized over the whole polymer chain, whereas for α,β-PTp the corresponding orbitals look rather localized. Especially in the area of the 2,5-bonds there is nearly no probability density of the orbitals, which suggests that this compound consists of basically conjugatively isolated dimeric units. This explanation is supported by the DOS plots in Figure 2. On the one hand, for PTp there are several broad populated areas, which indicate a relatively widespread conjugation over the polymer. But on the other hand, for α,β-PTp some small sharp areas of occupied states are visible, especially close to the Fermi edge. This implies flat energy bands in this area and is indicative of a relatively weak interaction between the unit cells [44]. In contrast, the DOS plot of PTp shows rather broad energy bands and, thus, a relatively strong intercellular interaction. The large band gap of α,β-PTp is not very favorable for most technical applications, hence we focus on PTp derivatives in the following.

### Influence of substituents

**Figure 4:**
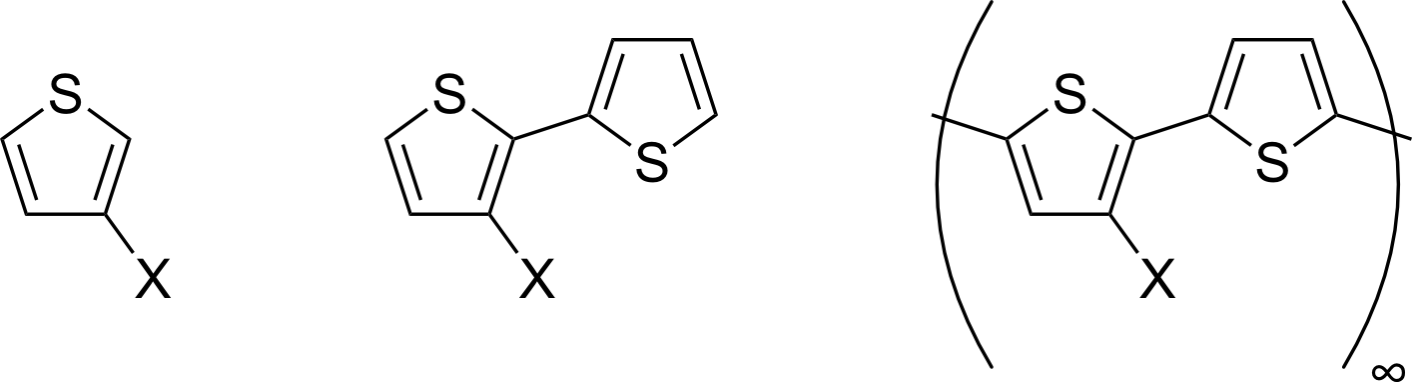
Illustration of the substitution patterns for singly bonded substituents of oligo- and polythiophenes considered in this study.

The main goal of this study is to determine how substituents affect the electronic properties of oligothiophenes and whether the underlying effects can be transferred to the respective polymers. First, we took into account *classical* substituents, namely methyl (CH_3_), amino (NH_2_) and nitro groups (NO_2_) and the chlorine atom (Cl). We chose these substituents, because they exemplify the basic electronic effects on the electronic charge distribution of conjugated systems known from organic chemistry. The considered substitution patterns for singly bonded substituents are illustrated in Figure 4. Furthermore, we have considered an annulated phenyl ring as a kind of special substituent to see how an explicitly extended π-system influences the respective systems.

Structural effects concerning bond lengths in the monomers and dimers compared to the unsubstituted Tp and BTp turned out to be negligibly small. Nevertheless, the dihedral angle between the two aromatic ring-systems in the substituted dimers differs from BTp. Except for the chlorine-substituted dimer (ClBTp), all BTp derivatives show dihedral angles of about 22*°* to 24*°*. ClBTp itself is predicted to appear in a totally flat structure, probably caused by the intramolecular dipole–dipole interaction. The already mentioned flattening effect of a growing chain length again becomes observable as the dihedral angles of the substituted polymers are about 12*°* for NO_2_PTp and NH_2_PTp, and the methyl- and chlorine-substituted polymers, such as PTp, turn out to be both completely flat.

The substituents lead to recognizable effects in the electronic structure of the oligothiophenes. As shown in Table 2, except for the methyl-substituted dimer, all substituted molecules reveal a lowered HOMO–LUMO gap. The nitro group definitely causes the largest effect among the considered substituents, lowering the gap by about 1*.*3 eV for the monomer and by about 0*.*7 eV for the dimer, which we tentatively assign to the strong negative mesomeric effect of the nitro group. The influence of all other considered substituents on the electronic structure is rather minor. Regarding the chlorine-substituted bithiophene, one should take its planar structure into account. Hence, the gap-lowering effect cannot solely be accredited to the direct electronic influence of chlorine. In addition, the steric effect has to be considered, as flat structures generally tend to form more stable conjugated systems and therefore smaller HOMO–LUMO gaps. Still, the fact that, with the exception of the nitro group, all considered constituents have a rather similar effect on the HOMO– LUMO gap in spite of the fact that they influence the dihedral angle in opposite ways, suggests that the geometry may not play such an important role in the electronic structure.

**Table 2:** Calculated HOMO–LUMO gaps *E*_g_ (in eV) for substituted oligothiophenes compared to the unsubstituted ones.

substituent	monomer	dimer

H	4.49	2.93
CH_3_	4.44	2.97
Cl	4.22	2.87
NH_2_	4.46	2.75
NO_2_	3.21	2.22

**Figure 5:**
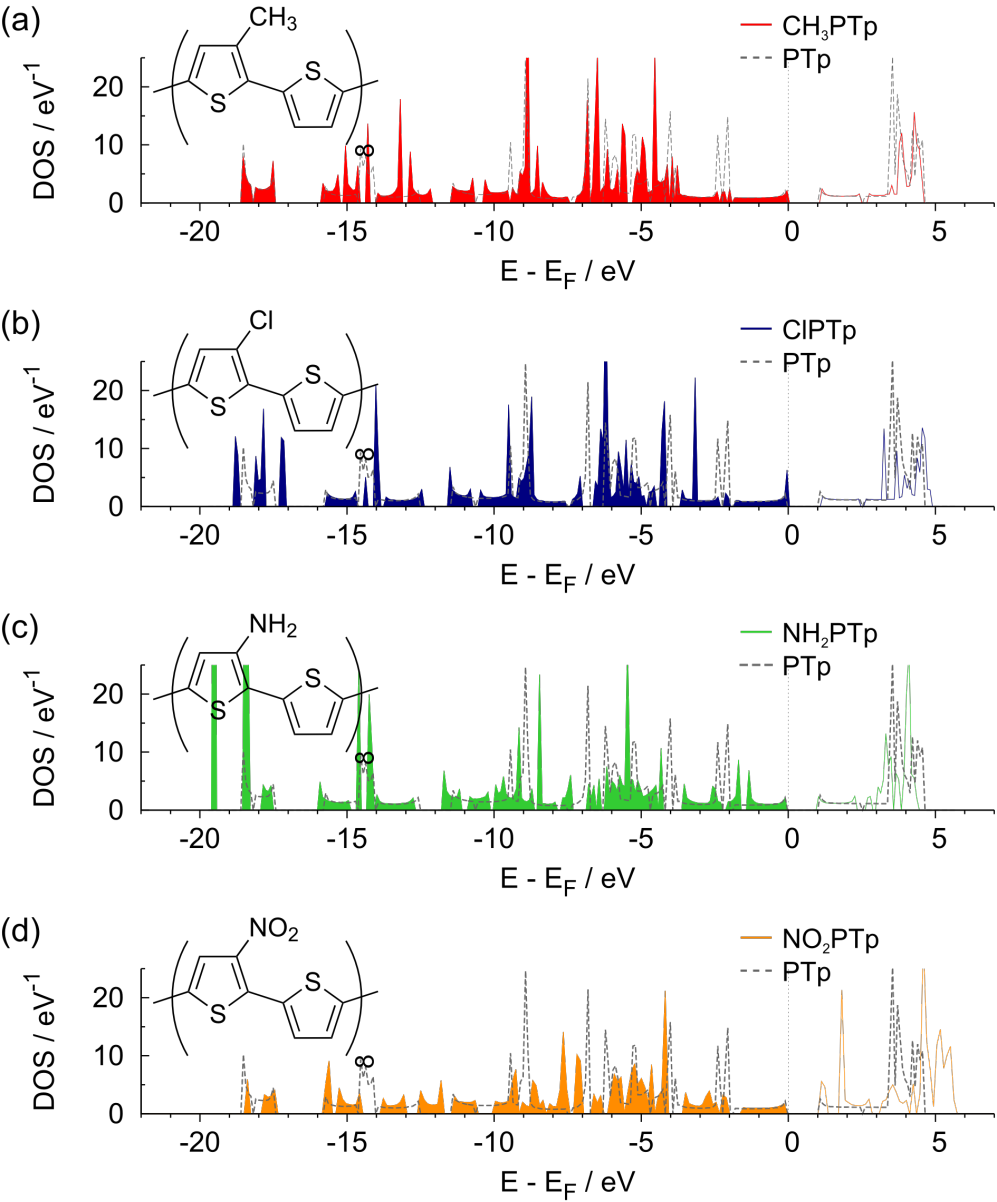
Density of states of substituted polymers: (a) CH_3_PTp, (b) ClPTp, (c) NH_2_PTp and (d) NO_2_PTp. As a comparison, in each panel the DOS of the unsubstituted PTp is indicated by the dashed lines.

In Figure 5, the resulting DOS of the substituted polymers is compared with the DOS of the unsubstituted PTp. Interestingly enough, although there are some changes in the band structure, there is only a minor effect of the substituents on the band gap. The band gap of 1*.*19 eV for the unsubstituted polythiophene is changed to 1*.*19 eV (CH_3_PTp), 1*.*22 eV (ClPTp), 1*.*14 eV (NH_2_PTp), and 1*.*27 eV (NO_2_PTp). The nitro group, which caused the largest reduction in the HOMO–LUMO gap for the monomer and dimer, now even leads to an increase of the band gap.

Hence, the influence of the substituents on the electronic structure is significantly reduced upon the transition from oligo- to polymer. This agrees with the results obtained by Salzner who reported similarly small effects of hydroxy and cyano substituents [15]. These groups lower the band gap of polymers only by about 0*.*1 eV, whereas they reduce the HOMO–LUMO gap of monomers by more than 1 eV.

**Figure 6:**
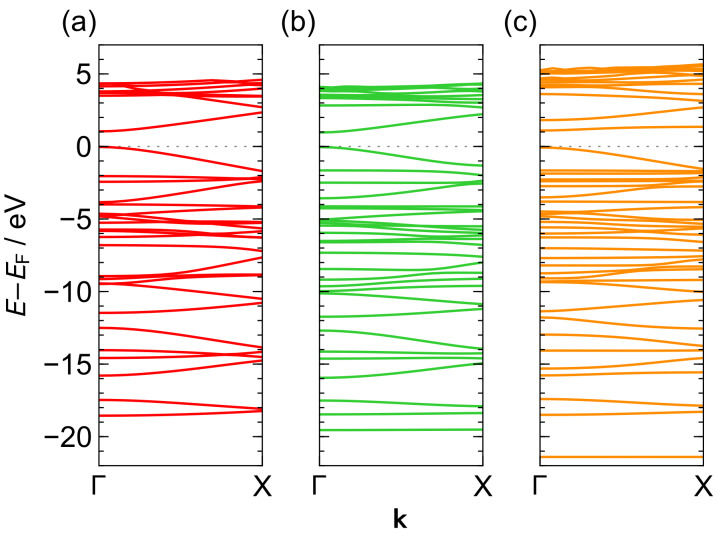
Calculated band structure of (a) unsubstituted PTp and of the substituted polymers (b) NH_2_PTp and (c) NO_2_PTp.

In order to analyze the reason for the rather similar band gaps, we compare in Figure 6 the band structures of the unsubstituted polymer PTp (Figure 6a) with the substituted polymers NH_2_PTp (Figure 6b) and NO_2_PTp (Figure 6c). The amino group does not change the band structure significantly. The substituted polymer is still a direct-band-gap semiconductor with the band gap located at the Γ-point. Apparently, the delocalized states at the band gap are only weakly disturbed by the presence of the amino group. Only far below the Fermi energy are some additional almost dispersionless bands visible, reflecting the existence of localized substituent states. These features are in fact present for all considered substituents, which lead to band structures that resemble the one of NH_2_PTp.

The only exception is NO_2_PTp whose band structure is shown in Figure 6c. A rather flat band related to the addition of the nitro group appears at about 1 eV above the valence band. This flat, almost dispersionless band indicates the presence of localized electronic states caused by the presence of the strongly interacting nitro group. The former conduction band of the unsubstituted polymer is shifted up by about another 1 eV. This demonstrates that the apparently only weak modification of the band gap upon substitution with the nitro group is only coincidence, since the substitution significantly modifies the band structure of the polymer.

**Figure 7:**
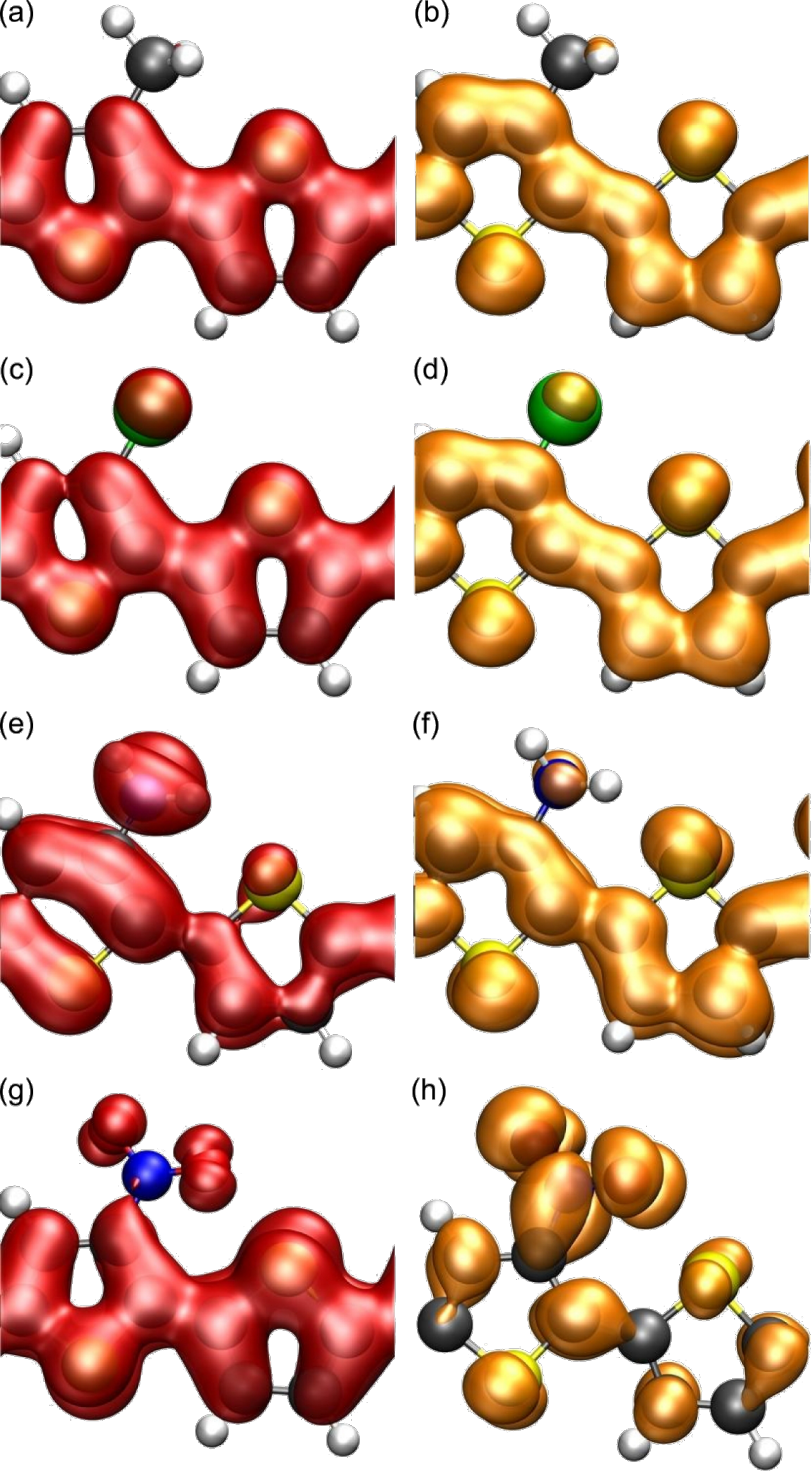
Electronic density isosurfaces (ρ(*r*) = 0*.*01 *e/*Å^3^) of the HOCO (red) and LUCO (orange) for (a), (b) CH_3_PTp; (c), (d) ClPTp; (e), (f) NH_2_PTp; (g), (h) NO_2_PTp.

One might assume that the small changes in the band gaps are a consequence of the fact that the substituents hardly affect the HOCO and LUCO. But this assumption can be rejected regarding Figure 7. There, the electronic density isosurfaces of the HOCO and the LUCO for the substituted polymers are shown, which should be compared to the corresponding plot of unsubstituted polymer in Figure 3. Also the amino group leads to significant changes in both the HOCO and the LUCO although it only caused minor changes of the band structure. It has been suggested that one assumes a similar energetic shift of both orbitals for π-donating/accepting substituents resulting in nearly unaltered values for the respective band gaps [45]. However, energetic shifts with respect to the vacuum level can be caused both by changes in the band structure as well as by the presence of modified dipole fields that arise due to the presence of the constituents. It is rather hard to disentangle these two contributions. Still it can be concluded that although the singly bonded substituents have some effect on the electronic structure of both oligomers and polymers, they hardly affect the band gap of the corresponding polymers. Only if the substituents are strongly interacting, such as the nitro group, do significant changes in the band structure and the orbitals result (see Figure 3g and 3h).

**Figure 8:**
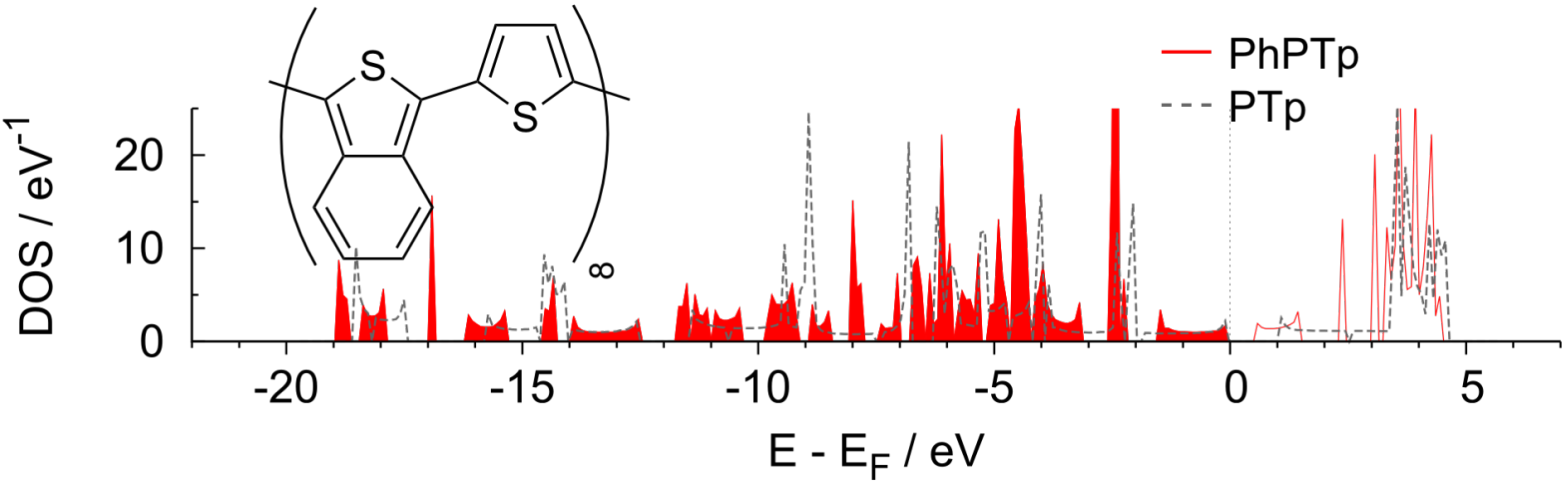
Calculated DOS of PhPTp compared to PTp. The inset illustrates the structure of PhPTp.

Until now we focused our investigation on classic substituents, which are all basically singly bonded to the aromatic ring system of the thiophene backbone. In order to extend our study, we considered a phenyl ring as a substituent, thus obtaining benzo[*c*]thiophen (PhTp), 1-(thiophen-3-yl)-benzo[*c*]thiophen (PhBTp) and the corresponding polymer (PhPTp, see below inset of Figure 8 for an illustration). Since this *π*-extending substituent differs significantly from those previously regarded in that it is bonded to two different carbon atoms of the thiophene backbone, we discuss it here separately.

Our calculations yielded a dihedral angle of about 34*°* for PhBTp and 21*°* for the corresponding polymer, respectively. Note that this is about twice the dihedral angle of NH_2_PTp and NO_2_PTp due to the steric demand of the annulated phenyl ring. Still, the previously observed flattening effect upon growing chain lengths also holds for this system. The HOMO–LUMO gap for PhTp is predicted to be 2*.*71 eV, which is far below the other substituted monomers discussed so far. This is reasonable because the annulated phenyl ring extends the conjugated π-system quite considerably. For the dimer, the calculated HOMO–LUMO gap is further reduced to 2*.*19 eV, which is rather close to the corresponding nitro-substituted analogue. However, in contrast to the polymers with singly bonded substituents, the PhPTp polymer exhibits a direct band gap of 0*.*7 eV that is also significantly reduced with respect to the unsubstituted polymer PTp, as Figure 8 shows. Apparently, the larger π-system of the phenyl-substituted polythiophene affects the electronic structure of polythiophene to a larger extent and leads to a smaller band gap. However, the valence band below the gap and the conduction band above the gap become narrower compared to the unsubstituted polymer (compare Figure 8 with Figure 2a) indicating more localized states. Note that Hong and Marynick found an increased direct band gap for an annulated cyclobutene ring [46], but also significantly reduced direct band gaps for other cyclic substituents. This suggests that it is possible to both increase and decrease the band gap with the choice of a suitable annulated substituent. Hence, annulated systems may be promising candidates for the manipulation of the band gap of polythiophene.

### Vinyl-bridged polythiophene derivatives

**Figure 9:**
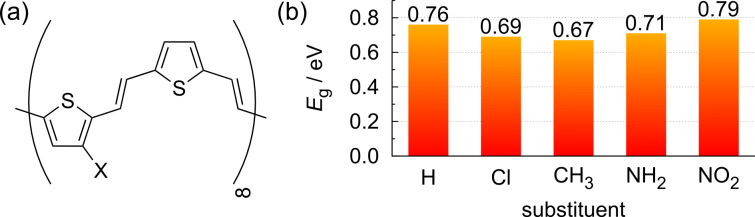
Considered vinyl-bridged polythiophene derivatives. (a) Structural formula, (b) band gaps *E*_g_ of the corresponding polymers.

In the discussion about the singly bonded substituents, we mentioned that the steric repulsion between the substituents also influences the geometric and electronic structure of the polythiophenes. In order to minimize this steric repulsion between the substituents, we considered polymers in which the thiophene rings in the backbone of the polymers are separated by a vinyl bridge (see Figure 9a). This results in entirely flat structures, independent of the respective substituent. Thus, geometric effects, such as deviations in the dihedral angle of the polymer, should not influence the band structure.

As Figure 9b demonstrates, the inclusion of a vinyl bridge also reduces the band gaps significantly from 1*.*2 eV for PTp to about 0*.*7 eV for the vinyl-bridged polymers. Apparently, the vinyl bridges reduce the aromaticity of the polymers by modifying the structure toward a quinoid form, leading to reduced band gaps, as the band gap of conjugated polymers depends (among other factors) on the degree of the quinoid or aromatic character of the backbone [20,46].

The trends among the substituents are similar to those for the substituted polythiophenes. Again, the nitro-substituted polymer reveals the largest band gap among the polymers. A closer look at the band structure and the density of states reveals that the widths of the bands are hardly modified, it is just the band gap between the valence and the conduction band that is reduced. Note that the band gap of the vinyl-bridged polymer with an annulated phenyl ring is even further decreased to 0*.*25 eV. Obviously, the effects of adding π-extending substituents and including vinyl bridges are roughly additive and can be combined in order to tailor the band gap.

### Influence of doping on the electronic structure

The electrical conductivity of a large class of polymers, in particular of polythiophene, can be highly increased when they are doped. The doping process itself corresponds basically to a manipulation of the number of valence electrons of the polymers, often in an electrochemical environment induced by adding counter ions. In order to model these doped compounds we varied the number of valence electrons per unit cell. Counter ions were not explicitly considered but modeled through a homogeneous charge background. Because polythiophene is known to be a good conductor in the p-doped state [11], we limited our study to oxidized states. Note that the exact nature of the charge carriers in doped polythiophenes is still debated, i.e., it is discussed whether the conductivity is caused by bipolarons or polaron pairs [18–19]. Since our unit cell only contains two aromatic rings, we cannot address polarons, which are supposed to extend over five thiophene rings [18]. Still, our results may be helpful to understand trends in the band gap engineering. Furthermore, we note that it has been shown that changing the oxidation state through electrochemical potential control can have a decisive influence on the conductivity of molecular junctions [47].

Table 3 lists calculated bond lengths for PTp in different oxidized states. When the polymer is neutral, a unit cell consisting of two thiophene rings contains 48 valence electrons. Obviously there are some bonds that lengthen and some bonds that contract when PTp is oxidized. A closer inspection reveals that the formerly short bonds lengthen and vice versa. All in all this results in a change into a quinoid-like structure that becomes more distinct the more the polymer is oxidized. This quasi-shift of the double bond goes along with a loss of aromaticity and thus should be energetically unfavorable at first glance. Of course the aromatic structure is more stable in the ground state, which is confirmed computationally [48], but the quinoid-like structure has a smaller ionization potential and a bigger electron affinity, and thus, the structural change caused by oxidation can be explained with the overall higher affinity of the quinoid-like structure towards charges [13].

**Table 3:** Calculated bond lengths for PTp (in Å) as a function of the charge state per unit cell in units of the elementary charge *|e|*.

charge state/unit cell^a^						
	2.0	1.0	0.8	0.5	0.3	0.0

C^1^–C^2^	1.45	1.43	1.42	1.41	1.40.	1.39
C^2^–C^3^	1.37	1.38	1.38	1.39	1.39	1.41
C^4^–C^6^	1.42	1.41	1.42	1.42	1.42	1.44
C^1^–S	1.75	1.73	1.74	1.74	1.74	1.74

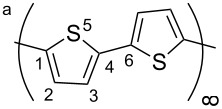

**Figure 10:**
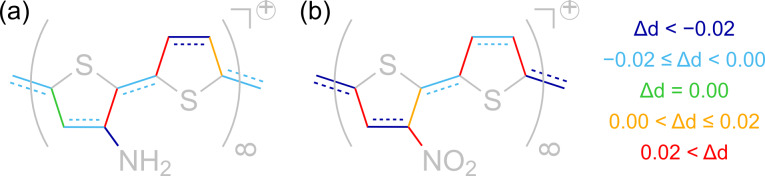
Color-coded change of bond lengths in (a) NH_2_PTp and (b) NO_2_PTp for positively charged polymers with the number of electrons per unit cell lowered by one.

Concerning substituted polymers, we have limited our investigation in this case to NO_2_PTp and NH_2_PTp as these two substituents are considered to have mesomeric effects, which are of special importance when it comes to (de)stabilization of excess charges. Regarding these polymer, the effects of doping are basically the same. Both reveal a tendency to form a quinoid-like structure in the oxidized state. However, as a consequence of the broken symmetry that comes along with the addition of a substituent, these quinoid-like structures are distorted to a certain extent. Figure 10 illustrates the color-coded change of the respective bond lengths in oxidized polymers.

Note that in the case of the amino-substituted polymer there is a contraction of the carbon-substituent bond by about 0*.*05 Å. In contrast, the corresponding bond length in NO_2_PTp increases by about 0*.*03 Å. This may be due to mesomeric effects. The nitro group is known to destabilize positive excess charges whereas the amino group usually stabilizes them through its +M-effect of organic chemistry, i.e., by its capacity to increase the electron density of the rest of the molecule. Hence, on the one hand, the NH_2_-group may shift electron density into the formerly aromatic electron-lacking ring system. On the other hand, it may be energetically favorable for an electron-lacking system to quit the conjugation to the nitro group and therefore to lengthen the respective bond. This could be a reason for the observed distortions of the polymer structure.

**Figure 11:**
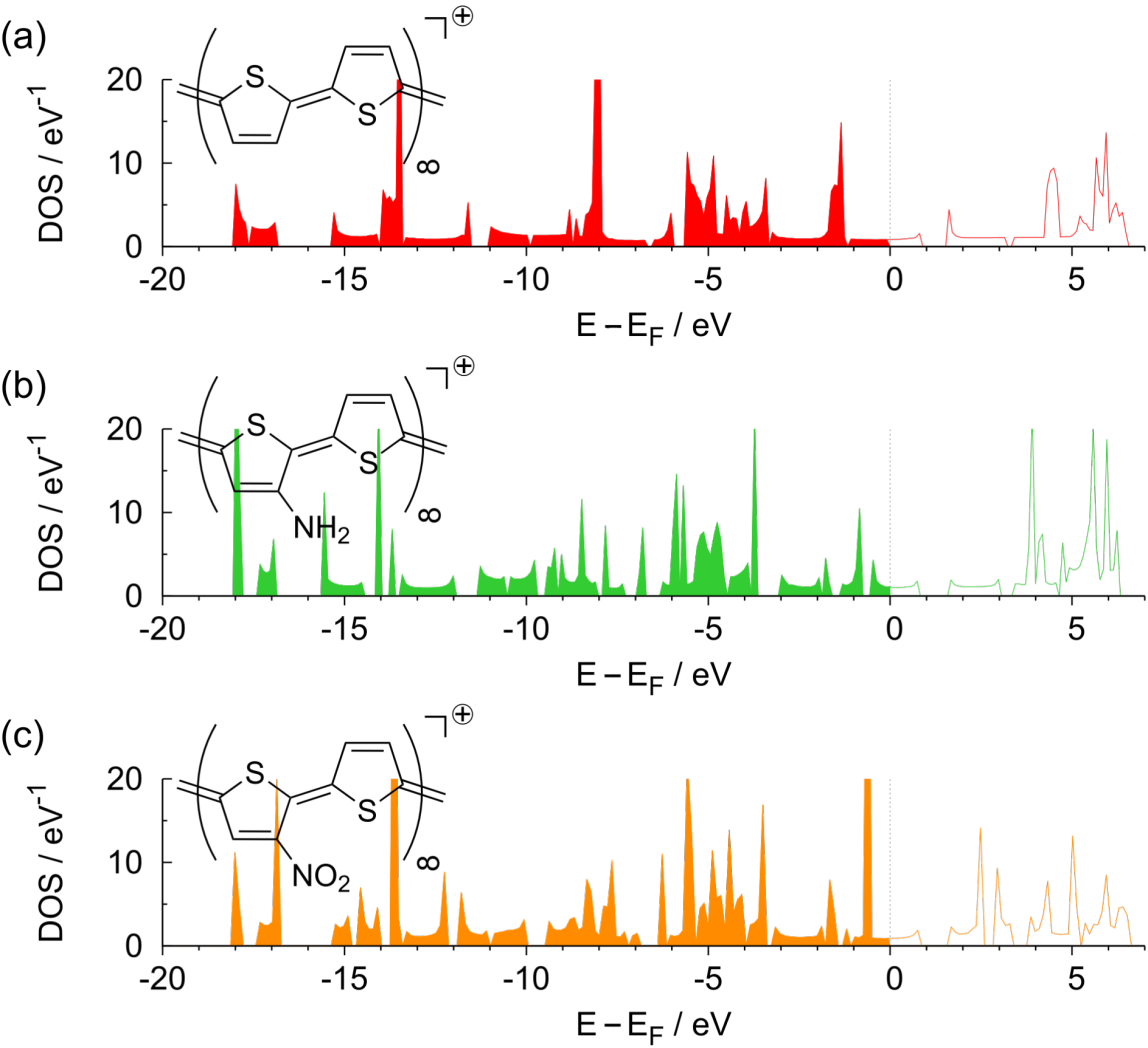
Density of states for positively charge polymers corresponding to a charge of 1*|e|* per unit cell: (a) PTp, (b) NH_2_PTp and (c) NO_2_PTp.

Regarding the density of states of the oxidized polymers plotted in Figure 11, it is obvious that positively charging the polymers leads to a partially occupied valence band, whereas the band structure is hardly changed compared to the neutral polymers. This indicates that charging the polymers basically corresponds to a shift of the Fermi energy without significant changes in the band structure and leads to metallic behavior. The substituted polymers, in contrast, still exhibit band gaps, cf. Figure 7. This means that the modification of the electronic structure upon substitution cannot be explained by simple electron removal or addition.

The resulting metallic state of the considered polymers seems to be at variance with the well-known fact that for π-conjugated organic polymers electrical conductivity cannot be understood with the mobility of unpaired electrons [48]. In fact, one-dimensional metals tend to distort spontaneously such that the spacing between adjacent unit cells becomes modulated [49]. In the case of polymers, conduction is associated with the formation of polarons or bipolarons. Quite often this leads to the formation of modulated quinoid-like structures [18–19] that extend over about five thiophene rings. In fact, as illustrated in Figure 10, we also find indications of a quinoid-like modification upon oxidizing the polymers. Yet, since our unit cell only contains at most two thiophene rings, such polarons, which would probably lead to the existence of a band gap, cannot be formed in our periodic DFT calculations. In order to address this issue, larger unit cells are required. Such calculations, which are more time-consuming, are planned for the future.

## Conclusion

The structural and electronic properties of oligo- and polythiophenes and their modifications through substituents have been studied by periodic density functional theory calculations. Whereas the considered oligothiophenes still exhibit nonvanishing dihedral angles, the corresponding polythiophenes turn out to be basically planar. Among the considered singly bonded substituents, methyl, amino or nitro groups, or a chlorine atom, the nitro group in particular leads to a significant modification of the HOMO–LUMO gap of thiophene monomers and dimers. In contrast, the corresponding polythiophenes exhibit a hardly modified band gap compared to the unsubstituted polythiophene.

Phenyl-substituted polythiophenes as well as vinyl-bridged polythiophene-derivatives, on the other hand, have drastically modified band gaps. In addition, positively charged polythiophenes were considered as a model for doped polythiophenes. All considered charged polythiophenes become metallic, which shows that the modified band gaps cannot be explained by simple electron removal or addition. However, the unit cell in the periodic DFT calculations was still too small to allow for the formation of polarons.
